# Characteristics of bioimpedance-determined fluid shifts according to intradialytic blood pressure difference

**DOI:** 10.1080/0886022X.2021.1988639

**Published:** 2021-10-20

**Authors:** Young Eun Kwon, Hye Min Choi, Dong-Jin Oh

**Affiliations:** Department of Internal Medicine, Division of Nephrology, Myongji Hospital, Hanyang University College of Medicine, Goyang, Republic of Korea

**Keywords:** Hemodialysis, fluid shifts, extracellular water, intracellular water, BP difference

## Abstract

This study was designed to identify the fluid spaces that are most changed during ultrafiltration (UF) according to intradialytic blood pressure (BP) difference. BP data were collected five times (before hemodialysis [HD] and 1–4 h of HD). Intradialytic BP difference was calculated as the highest minus lowest of these BP measurements. Intradialytic systolic BP (SBP) difference over 20 mm Hg and diastolic BP (DBP) difference over 10 mm Hg were defined as wide intradialytic SBP difference (SYS-W) and DBP difference (DIA-W), respectively. We measured the various fluid spaces before HD and 1–4 h of HD, and 30 min after HD using a portable, whole-body bioimpedance spectroscopy (BIS). In this study, 85 prevalent patients aged over 18 years with a fixed dry weight (65.38 ± 12.45 years, 54.18% men, 52.50% patients with diabetes), undergoing HD had participated. 1) Mean relative reduction of extracellular water (ECW) was significantly higher in SYS-W than in narrow intradialytic SBP difference (SYS-N) patients from 1 h to 30 min after HD. 2) Mean relative reduction of intracellular water (ICW) was significantly lower in DIA-W than in narrow intradialytic DBP difference (DIA-N) patients from 1 h to 30 min after HD. 3) ECW of patients with SYS-W was significantly lower than that of patients with SYS-N. Patients with SYS-W have the characteristics of fluid shifts in which reduction of ECW was steeper than patients with SYS-N whereas fluid shifts of ICW were lower in patients with DIA-W than patients with DIA-N.

## Introduction

A recent large, retrospective cohort study observed first-year mortality to be higher in HD patients with BP variability than in those with stable BP, independent of the absolute BP level, suggesting that BP variability is associated with worse outcomes at all levels of BP [1]. The majority of BP variability studies in the general and HD populations have considered long-term, visit-to-visit BP variability measured at clinical visits across days, weeks, or months [2,3]. Recent data suggest that short-term BP variability, considered as intradialytic BP fluctuations, is a cardiovascular risk factor among HD patients [4].

Fluid shifts are commonplace in chronic HD patients during the intradialytic periods. During UF, several liters of fluid are removed [5]. Existing literatures support the idea of reduction of extracellular (EC) fluid space during renal replacement therapy, confirmed with multiple techniques [6–9]. About two decades ago, the portable whole-body multi-frequency bioimpedance spectroscopy (BIS; body composition monitor [BCM]), which utilizes a well-established technical method for volume assessment [10–12], were developed and can be accessible to most dialysis units. Recently, there were two reports to investigate fluid shifts in hemodynamic-unstable patients during dialysis [13,14]. However, there are few literatures to describe the differential (EC *vs.* intracellular [IC]) source of fluid shifts during the UF procedure and the relative ratio of these compartments during conventional renal replacement therapy in patients with asymptomatic intradialytic BP variability. An authoritative journal accepted the definition of BP variability by the difference between the highest and lowest BP during each session [15]. Therefore, this study was designed to identify the fluid spaces that are most changed during ultrafiltration (UF) according to intradialytic blood pressure (BP) difference, but without symptomatic intradialytic hypotension (IDH), intradialytic hypertension (IH).

## Patients and methods

### Patient characteristics

In this study, 85 prevalent patients aged over 18 years with a fixed dry weight (65.38 ± 12.45 years, 54.18% men, 52.50% patients with diabetes), undergoing HD had participated. Patients who were hemodynamically unstable [SBP < 90 mmHg or SBP < 100 mmHg with symptoms of nausea/vomiting or trendelenburg position or intradialytic fluid infusion to normalize BP during HD], patients with a history of ischemic or hemorrhagic stroke, or heart disease including atrial fibrillation (any ischemic and arrhythmic evidences by electrocardiogram, left ventricle ejection fraction (EF) < 45% by 2-D echocardiography); those with peripheral arterial occlusive disease, such as gangrene, and amputees; and those with infection or hemiplegia were excluded. Some of the patient’s characteristics are given in Table 1. Informed written consent was obtained from all patients. This study was approved by the local research ethics committee [MJH 2018-10-013].

**Table 1.** Characteristics of the study population (*n* = 85).

Variables	Value
Age (years)	65.38 ± 12.45
Males (%)	54.12
DM (%)	52.50
HD vintage (Mos)	70 (11–130)
Kt/V_urea_	1.51 (1.30–1.72)
EF (%)	59.03 ± 6.64
Relative OH (%)	15.29 ± 7.84
Pre-HD weight(kg)	62.30 ± 11.36
Inter-HD weight gain (kg)	2.16 ± 0.94
Pre-HD SBP (mmHg)	153.42 ± 21.49
Pre-HD DBP (mmHg)	69.13 ± 13.86
UFV (ml)	2457.65 ± 947.73
V_urea_ (L)	29.71 ± 6.92
TBW (L)	32.37 ± 7.32
ECW (L)	15.80 ± 3.21
ICW (L)	16.57 ± 4.39
E/I ratio	0.97 ± 0.13
SBP difference (mmHg)	32.66 ± 18.62
DBP difference (mmHg)	13.16 ± 7.05
Number of anti-hypertensive drugs	2 (1–4)

Data are expressed as the mean ± standard deviation (SD) or the median (range), count (%), respectively. DM: diabetes mellitus; HD: hemodialysis; Kt/V_urea_: urea clearance; EF: ejection fraction; OH: overhydration; SBP: systolic blood pressure; DBP: diastolic blood pressure; UFV: ultrafiltration volume; V_urea_: urea distribution volume; TBW: total body water; ECW: extracellular water; ICW: intracellular water; E/I ratio: ECW to ICW ratio

### Dialysis technique

All patients underwent high-flux HD thrice weekly with a bicarbonate buffer for 4 h, using 1.0–1.4-m^2^ hollow fiber poly-sulfone membranes, blood flow rates of 250–280 mL/min, and a dialysate flow rate of 500 mL/min, using the Fresenius 5008S machine (Bad Homburg, Germany). The mean UF volume was 2457.65 ± 947.73 mL, and treatment time was 4 h. The dialysis fluid contained 138 mEq/L sodium, 2 mEq/L potassium, 2.5 mEq/L calcium, 1.0 mEq/L magnesium, 108.5 mEq/L chloride, 35 mEq/L bicarbonate, and 99.1 mg/dL glucose, and the temperature was maintained at 36 °C.

### Definition of BP difference

BP data were collected five times (before HD and 1–4 h of HD), and the highest and lowest measurements were identified. Intradialytic BP difference was calculated as the highest minus lowest BP measurements of each dialysis session [15]. Intradialytic SBP difference over 20 mm Hg and DBP difference over 10 mm Hg were defined as wide intradialytic SBP difference (SYS-W) and wide DBP difference (DIA-W), respectively. On the contrary, intradialytic SBP difference under 20 mmHg and DBP difference under 10 mmHg were defined as narrow intradialytic SBP difference (SYS-N) and narrow DBP difference (DIA-N). In addition, patients with SYS-N and DIA-N are expressed as SYS-N/DIA-N patients whereas patients with SYS-W and DIA-W are classified as SYS-W/DIA-W patients. We also studied the fluid shifts between SYS-N/DIA-N and SYS-W/DIA-W patients. BP recordings were obtained by the dialysis unit staff in the patient’s supine position with a supported arm using an aneroid sphygmomanometer and the auscultatory technique. The BP was measured again, and the average of the two readings was recorded.

### Body composition monitoring

BCM measurement has been recommended that subjects should be resting in a supine position for at least 5 min, before the BCM-measurement is started, such that fluid volume equilibration has taken place [14]. We measured the various fluid spaces before HD and 1–4 h of HD, and 30 min after HD using a portable, whole-body ankle-to-wrist BIS (BCM; Fresenius AG software version 3.2; Bad Homburg, Germany) [16] at mid-week (Wednesday–Thursday). The BCM instrument returns measurements on overhydration (OH), TBW, ECW, ICW, urea distribution volume (V_urea_) in liters (L). The BCM determines resistance and reactance at 50 discrete frequencies from 3 to 1000 kHz. ECW and ICW resistance are obtained on the basis of a Cole model [17]. Using these read-out values, ECW, ICW, and TBW are automatically calculated by the BCM-device, employing a fluid model described by Moissl et al. [18].

### Statistical analysis

Because of the small number of patients, normal distribution was tested using a single sample Kolmogorov–Smirnov analysis. Continuous variables are expressed as mean ± SD or median (range) according to normal distribution of data. Categorical variables are expressed as percentage (%). Between-group differences were assessed for significance using a Student t-test for normal distribution, Mann–Whitney U tests for skewed distribution, or Chi-Square test for categorical variables. Statistical analyses were performed using SPSS software version 14.0 (SPSS Inc., Chicago, IL).

## Results

### Baseline characteristics

The study included a total of 85 patients undergoing HD (vintage of 70.98 ± 59.47 months), 46 (54.12%) men with a mean age of 65.38 ± 12.45 years. Here, 44 (51.76%) patients had diabetes mellitus and 50 (58.82%) patients had hypertension. The anti-hypertensive medications administered by the patients included angiotensin-converting enzyme inhibitor by nine patients (10.59%), angiotensin II receptor blocker by 18 patients (21.18%), calcium channel blocker by 30 patients (35.29%), alpha blocker by three patients (3.53%), beta-blocker by 21 patients (24.71%), diuretic by seven patients (8.24%), and other anti-hypertensive medications by 2 patient (2.35%). In 62 (72.94%) or 35 (41.17%) of the patients on HD, phosphorus binders or active vitamin D metabolites (cinacalcet and/or paricalcitol) were administered, respectively. Other variables are summarized in Table 1.

### Time course changes of fluid compartments according to intradialytic SBP difference

**Figure 1.**
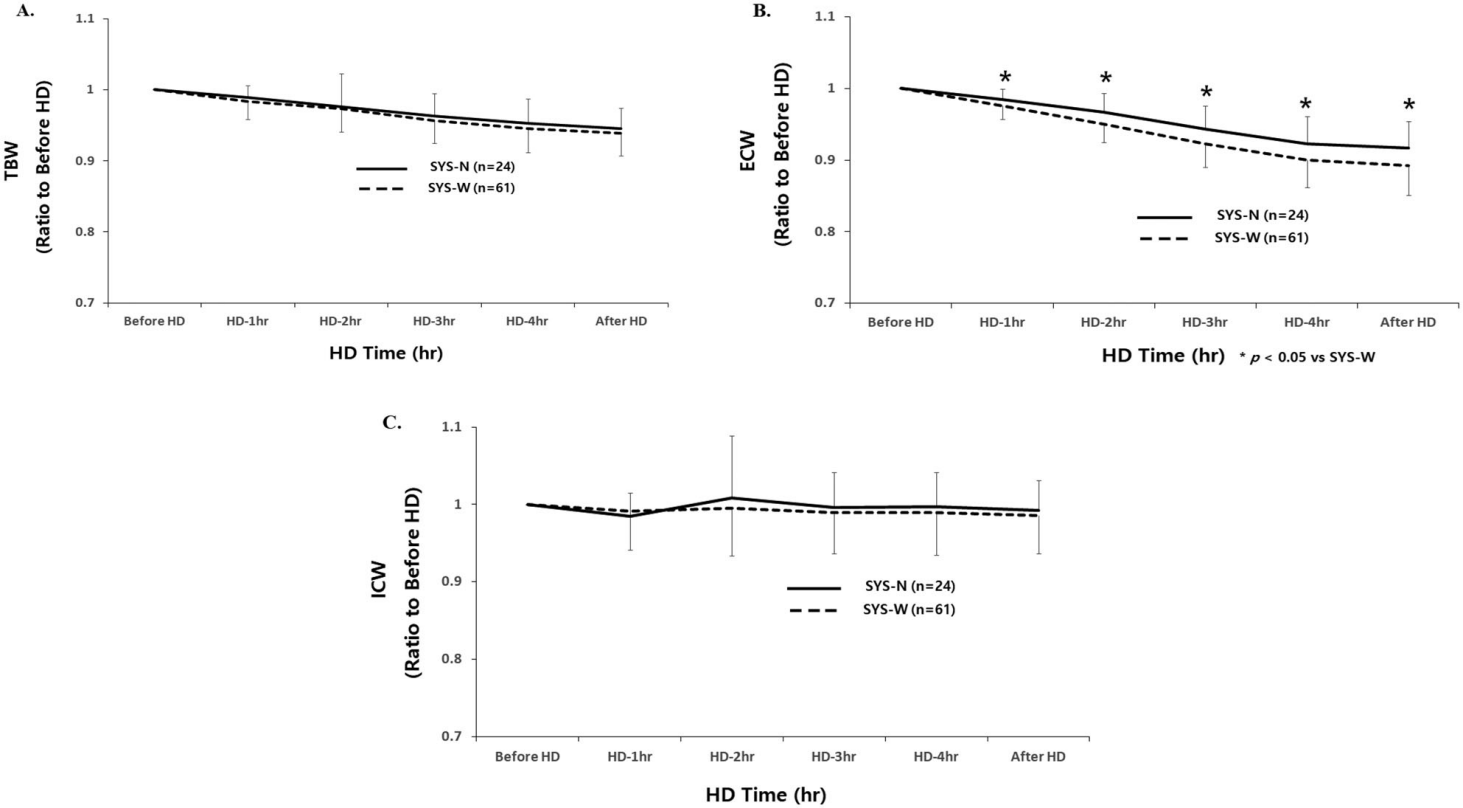
(A–C) Time course changes of fluid compartments according to intradialytic SBP difference. SYS-N: narrow intradialytic SBP difference; SYS-W: wide intradialytic SBP difference; TBW: total body water; ECW: extracellular water; ICW: intracellular water; HD: hemodialysis.

Mean relative reduction of TBW was not significantly different between SYS-N and SYS-W patients from the start to 30 min after HD (before HD, 1.00; 1 h, 0.984 ± 0.017 *vs.* 0.984 ± 0.027; 2 h, 0.988 ± 0.047 *vs.* 0.975 ± 0.034; 3 h, 0.970 ± 0.032 *vs.* 0.958 ± 0.032; 4 h, 0.960 ± 0.035 *vs.* 0.948 ± 0.035, 30 min after HD; 0.955 ± 0.029 *vs.* 0.941 ± 0.032, respectively) (Figure 1(A)).

Mean relative reduction of ECW was significantly higher in SYS-W than in SYS-N patients from 1 h to 30 min after HD (before HD, 1.00; 1 h, 0.984 ± 0.014 *vs.* 0.977 ± 0.019*; 2 h, 0.966 ± 0.027 *vs.* 0.952 ± 0.026*; 3 h, 0.942 ± 0.032 *vs.* 0.924 ± 0.033*; 4 h, 0.923 ± 0.037 *vs.* 0.901 ± 0.038*, 30 min after HD; 0.916 ± 0.038 *vs.* 0.895 ± 0.042*, respectively, *p < .05) (Figure 1(B)).

Mean relative reduction of ICW was not significantly different between SYS-N and SYS-W patients from the start to 30 min after HD (before HD, 1.00; 1 h, 0.984 ± 0.031 *vs.* 0.991 ± 0.052; 2 h, 1.008 ± 0.081 *vs.* 0.996 ± 0.064; 3 h, 0.996 ± 0.047 *vs.* 0.989 ± 0.054; 4 h, 0.997 ± 0.044 *vs.* 0.991 ± 0.058, 30 min after HD; 0.992 ± 0.038 *vs.* 0.987 ± 0.049, respectively) (Figure 1(C)).

### Time course changes of fluid compartments according to intradialytic DBP difference

**Figure 2.**
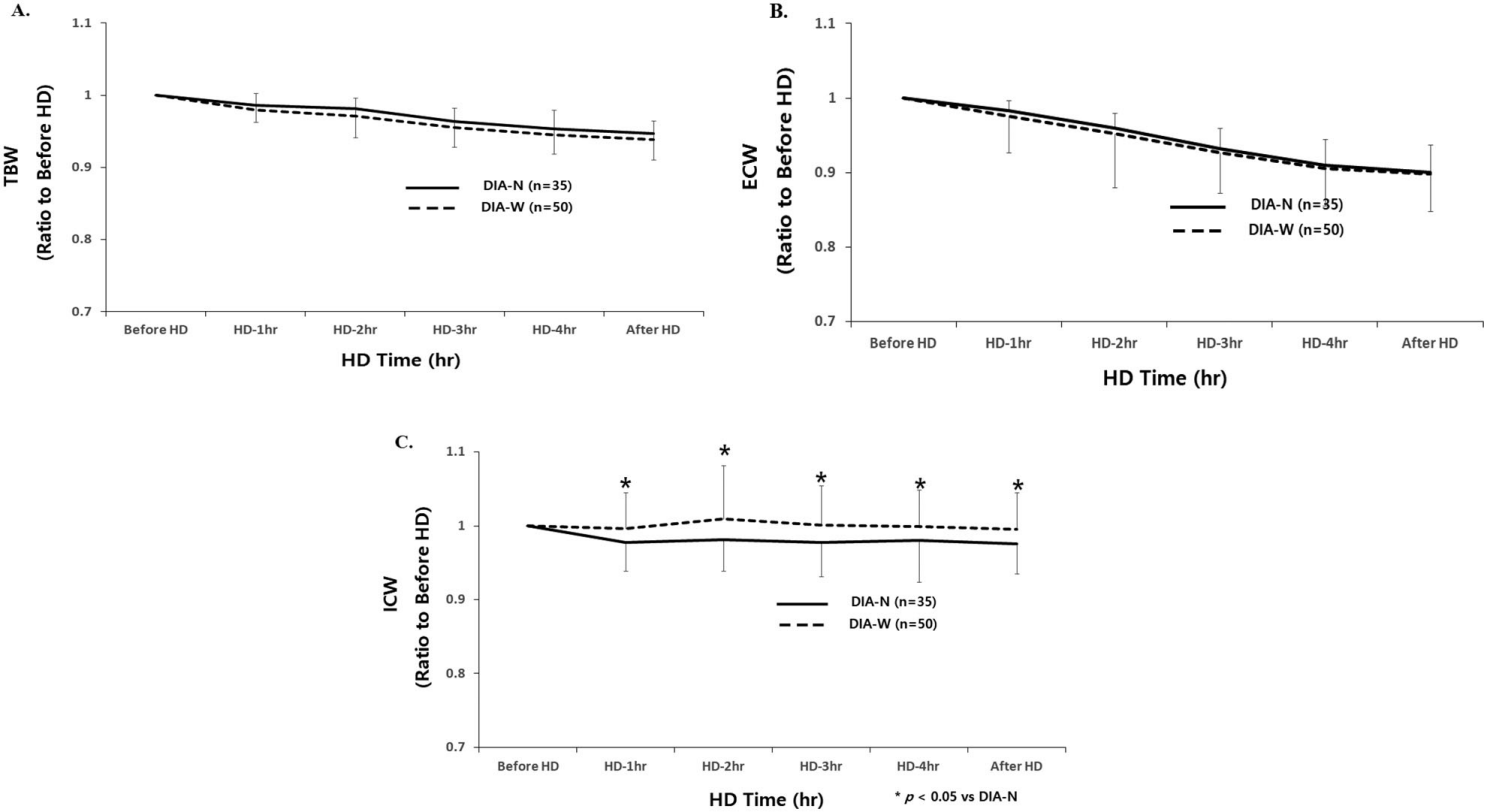
(A–C) Time course changes of fluid compartments according to intradialytic DBP difference. DIA-N: narrow intradialytic DBP difference; DIA-W: wide intradialytic DBP difference; TBW: total body water; ECW: extracellular water; ICW: intracellular water; HD: hemodialysis.

Mean relative reduction of TBW was not significantly different between DIA-N and DIA-W patients from the start to 30 min after HD (before HD, 1.00; 1 h, 0.980 ± 0.023 *vs.* 0.986 ± 0.024; 2 h, 0.971 ± 0.025 *vs.* 0.982 ± 0.040; 3 h, 0.955 ± 0.027 *vs.* 0.964 ± 0.036; 4 h, 0.945 ± 0.034 *vs.* 0.953 ± 0.036, 30 min after HD; 0.938 ± 0.026 *vs.* 0.947 ± 0.036, respectively) (Figure 2(A)).

Mean relative reduction of ECW was not significantly different between DIA-N and DIA-W patients from start to 30 min after HD (before HD, 1.00; 1 h, 0.983 ± 0.014 *vs.* 0.975 ± 0.021; 2 h, 0.959 ± 0.021 *vs.* 0.953 ± 0.030; 3 h, 0.932 ± 0.027 *vs.* 0.926 ± 0.038; 4 h, 0.910 ± 0.035 *vs.* 0.905 ± 0.042, 30 min after HD; 0.901 ± 0.036 *vs.* 0.898 ± 0.046, respectively) (Figure 2(B)).

Mean relative reduction of ICW was significantly lower in DIA-W than in DIA-N patients from 1 h to 30 min after HD (before HD, 1.00; 1 h, 0.977 ± 0.038 *vs.* 0.997 ± 0.049*; 2 h, 0.982 ± 0.043 *vs.* 1.009 ± 0.073*; 3 h, 0.977 ± 0.045 *vs.* 1.001 ± 0.053*; 4 h, 0.980 ± 0.056 *vs.* 0.999 ± 0.050*, 30 min after HD; 0.975 ± 0.040 *vs.* 0.995 ± 0.050, respectively, *p < .05) (Figure 2(C)).

### Time course changes of fluid compartments according to intradialytic SBP and DBP difference

**Figure 3.**
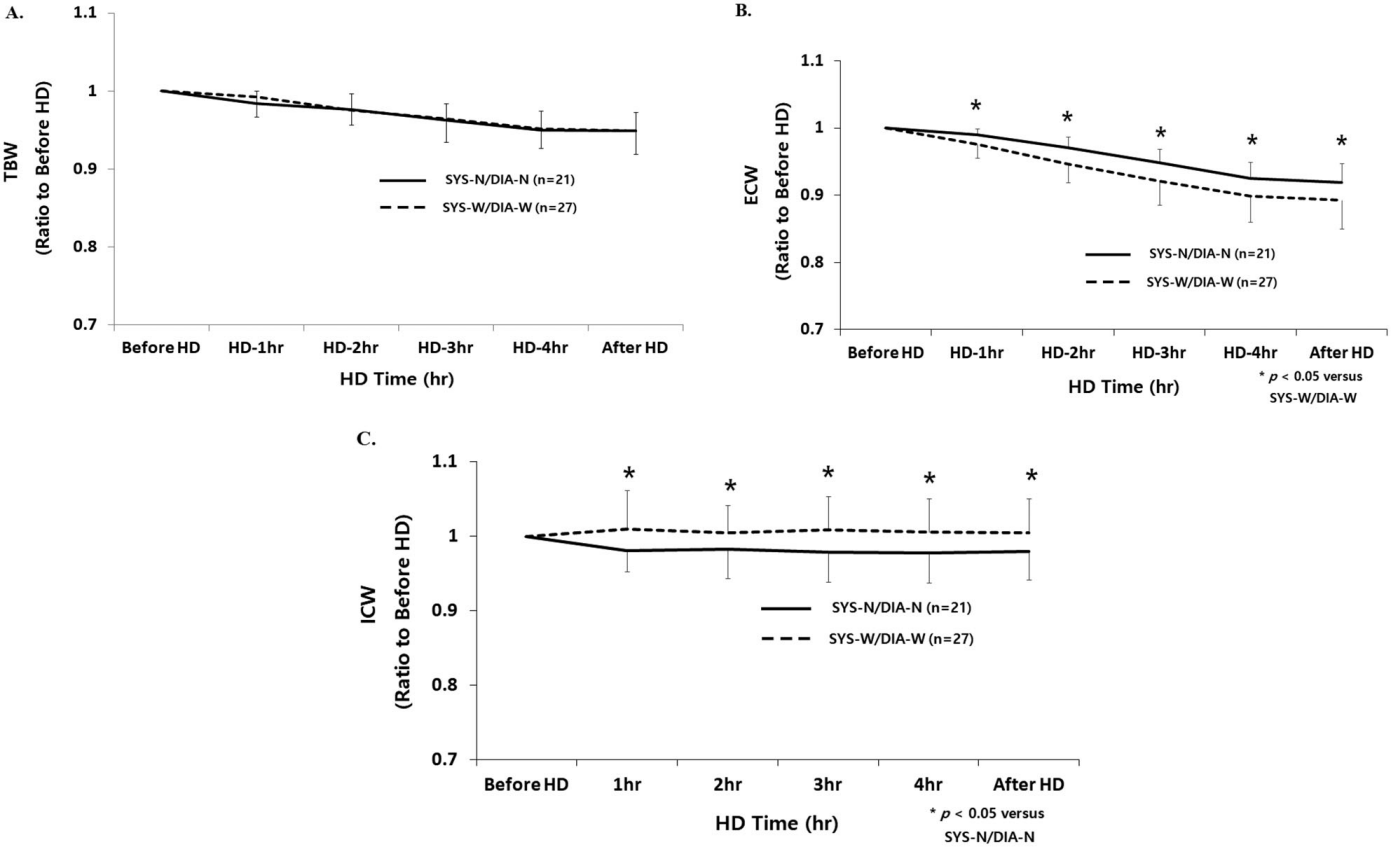
(A–C) Time course changes of fluid compartments according to intradialytic SBP and DBP difference. SYS-N/DIA-N: narrow intradialytic SBP-W/DIA-W: wide intradialytic SBP/DBP difference; TBW: total body water; ECW: extracellular water; ICW: intracellular water; HD: hemodialysis.

Mean relative reduction of TBW was not significantly different among patients with SYS-N/DIA-N and those with SYS-W/DIA-W from the start to 30 min after HD (before HD, 1.00; 1 h, 0.982 ± 0.016 *vs.* 0.993 ± 0.021; 2 h, 0.977 ± 0.021 *vs.* 0.977 ± 0.016; 3 h, 0.965 ± 0.024 *vs.* 0.966 ± 0.029; 4 h, 0.953 ± 0.020 *vs.* 0.953 ± 0.025, 30 min after HD; 0.950 ± 0.021 *vs.* 0.950 ± 0.030, respectively) (Figure 3(A)).

Mean relative reduction of ECW was significantly lower in patients with SYS-N/DIA-N and those with SYS-W/DIA-W from 1 h to 30 min after HD (before HD, 1.00; 1 h, 0.989 ± 0.009 *vs.* 0.977 ± 0.019*; 2 h, 0.969 ± 0.014 *vs.* 0.949 ± 0.026*; 3 h, 0.946 ± 0.018 *vs.* 0.923 ± 0.034*; 4 h, 0.924 ± 0.018 *vs.* 0.904 ± 0.038*, 30 min after HD; 0.922 ± 0.019 *vs.* 0.885 ± 0.043*, respectively, *p < .05) (Figure 3(B)).

Mean relative reduction of ICW was significantly higher in patients with SYS-N/DIA-N and those with SYS-W/DIA-W from 1 h to 30 min after HD (before HD, 1.00; 1 h, 0.977 ± 0.030 *vs.* 1.014 ± 0.050*; 2 h, 0.983 ± 0.039 *vs.* 1.010 ± 0.034*; 3 h, 0.982 ± 0.042 *vs.* 1.015 ± 0.037*; 4 h, 0.982 ± 0.034 *vs.* 1.012 ± 0.038*, 30 min after HD; 0.984 ± 0.036 *vs.* 1.010 ± 0.039*, respectively, *p < .05) (Figure 3(C)).

### Comparison of clinical parameters according to intradialytic BP difference

ECW of patients with SYS-W was significantly lower than that of patients with SYS-N. Other variables including age, sex, presence of DM, HT medication, HD vintage, EF, relative OH, pre-HD weight, normohydration (NH) weight, UFV, V_urea_, TBW, ICW, and E/I ratio were no significant difference between the groups (Table 2).

**Table 2.** Comparison of clinical parameters according to intradialytic BP difference.

	SYS-N (*n* = 24)	SYS-W (*n* = 61)	DIA-N (*n* = 35)	DIA-W (*n* = 50)	SYS-N/DIA-N (*n* = 21)	SYS-W/DIA-W (*n* = 27)
Age (year)	68.93 ± 10.88	65.86 ± 13.13	66.97 ± 11.63	65.83 ± 13.15	68.69 ± 12.90	65.81 ± 12.67
Male (%)	49	55	55	54	54	52
Presence of DM (%)	48	55	46	52	46	59
CCB (%)	9/24 (37.50%)	21/61 (34.43%)	12/35 (34.29%)	18/50 (36.00%)	8/21 (38.09%)	10/27 (37.04%)
ARB or ACE inhibitors (%)	8/24 (33.33%)	19/61 (31.15%)	11/35 (31.43%)	16/50 (32.00%)	6/21 (28.57%)	8/27 (29.63%)
β-blocker (%)	6/24 (25.00%)	15/61 (24.59%)	9/35 (25.71%)	12/50 (24.00%)	5/21 (23.81%)	7/27 (25.93%)
HD vintage (Mos)	69 (11–129)	70 (11–130)	70 (11–129)	70 (12–130)	70 (11–130)	70 (11–129)
EF (%)	58.51 ± 5.75	59.53 ± 5.50	59.35 ± 4.25	58.26 ± 5.48	60.10 ± 3.10	58.55 ± 7.58
Relative OH (%)	16.23 ± 4.99	14.52 ± 5.80	15.84 ± 5.41	15.02 ± 5.67	14.00 ± 10.17	14.44 ± 6.79
Pre-HD weight (kg)	64.69 ± 11.78	60.79 ± 10.26	62.69 ± 9.07	60.08 ± 13.68	60.60 ± 9.17	59.79 ± 11.44
NH weight (kg)	61.90 ± 11.39	58.53 ± 10.09	60.10 ± 8.97	58.73 ± 11.58	58.23 ± 8.79	57.57 ± 11.19
UFV (mL)	2255.56 ± 922.51	2536.73 ± 810.01	2348.39 ± 712.68	2452.38 ± 888.75	2476.92 ± 996.79	2377.78 ± 936.99
V_urea_ (L)	31.41 ± 6.71	28.85 ± 6.40	30.14 ± 6.13	29.12 ± 7.09	28.79 ± 5.74	28.12 ± 6.34
TBW (L)	33.67 ± 6.60	31.39 ± 6.66	32.58 ± 6.13	31.57 ± 7.22	32.67 ± 7.68	30.50 ± 6.83
ECW (L)	16.82 ± 3.11	15.43 ± 2.85*	16.16 ± 2.56	15.61 ± 3.34	15.98 ± 3.35	15.08 ± 3.08
ICW (L)	16.51 ± 3.65	15.94 ± 4.00	16.40 ± 3.75	15.97 ± 4.10	16.66 ± 4.70	15.42 ± 3.98
E/I ratio	1.01 ± 0.10	1.00 ± 0.12	1.00 ± 0.11	1.00 ± 0.12	0.98 ± 0.15	1.00 ± 0.14

Data are expressed as the mean ± standard deviation (SD) or the median (range), count (%), respectively. SYS-N: narrow intradialytic SBP difference (≤ 20 mmHg); SYS-W: wide intradialytic SBP difference (> 20 mmHg); DIA-N: narrow intradialytic DBP difference (≤ 10 mmHg); DIA-W: wide intradialytic DBP difference (> 10 mmHg); SYS-N/DIA-N: narrow intradialytic SBP and DBP difference; SYS-W/DIA-W: wide intradialytic SBP and DBP difference; DM: diabetes mellitus; CCB: calcium channel blocker; ARB: angiotensin receptor blockade; ACE: angiotensin-converting enzyme; HD: hemodialysis; EF: ejection fraction; OH: overhydration; NH: nomohydration; UFV: ultrafiltration volume; V_urea_: urea distribution volume; TBW: total body water; ECW: extracellular water; ICW: intracellular water; E/I ratio: ECW to ICW ratio

*p < .05.

## Discussion

The main findings of our study are 1) changes in the ECW of patients with wide intradialytic SBP difference were significantly steeper than those of patients with narrow intradialytic SBP difference from 1 h to 30 min after HD. 2) there were no significant fluid shifts in the ICW of patients with wide intradialytic DBP difference, whereas that of patients with narrow intradialytic DBP difference was significantly decreased from 1 h to 30 min after HD. 3) ECW of patients with wide intradialytic SBP difference was significantly lower than that of patients with narrow intradialytic SBP difference.

The results of the study suggest that maintenance of intradialytic SBP stability is dependent on the amount of ECW reduction during HD and that DBP stability is associated with the shifts of ICW. That is because there was no difference in the changes of TBW, whereas the difference in ECW or ICW changes could be observed between patients with narrow SBP difference and wide SBP difference, between patients with narrow DBP difference and wide DBP difference during HD, respectively. This phenomenon has been confirmed from patients with narrow intradialytic SBP/DBP difference, once again. In particular, ICW in patients with narrow intradialytic SBP/DBP difference showed a significant decrease, whereas that in patients with wide intradialytic SBP/DBP difference demonstrated no significant reduction of ICW from 1 h of HD compared to the start of HD. The above changes were continued until 30 min after HD without further reduction or increase. This indicates that the amount of shifts of ICW to ECW may affect the maintenance of SBP and DBP stability in situations where ECW is continuously reduced after HD. Thus, in contrast to a previous report [19], fluid removed from the IC space would be expected to contribute to overall BP stability in our results. However, our study does not explain how the fluid shift from ICW to ECW can be distributed between the intravascular fluid (IVF) and interstitial fluid (ISF).

A recently introduced equipment called a BCM utilizes a well-established technical solution to assess the absolute volume of the body fluid distribution (ECW, ICW, and TBW) using 50 multiple, discrete frequencies from 3 to 1000 kHz [16]. Despite the above advantages, the BCM cannot elucidate the changes of IVF and ISF absolute values during HD. In addition, this device was originally designed to establish the fluid volume status in patients with dialysis patients, thereby helping the optimal fluid removal during the dialysis session [20]. Finally, the BCM is a whole-body BIS device. The precision of the method has been questioned in several research articles because of non-homogenic nature of human body [21–24]. Recent studies have shown that sum of segmental BIS is less affected by the change of body position and may be more accurate in measuring ECF change than whole body BIS [21–24]. Another problems lies in fluid transfer from limbs to trunk while turning the body position [22]. So, our results may be valid only when the same instrumentation and protocol are used and may only be valid for the test patients and researchers from the host facility. This article is more focused upon the use of bioimpedance in the clinical setting rather than as a research investigation to define mechanisms and underlying fluid responses to dialysis. We have, potentially, made our point that fluid responses are associated in some way to patient BPs as a factor between stable and unstable patients.

The clinical dilemmas and prognostic uncertainties exist in a patient with asymptomatic intradialytic BP falls, elevations, and fluctuations compared to overt intradialytic BP abnormalities such as IDH or IH. This absence of associated symptoms contributes to the tendency to regard asymptomatic BP fluctuations as ‘normal’ BP. However, there is a possibility that aberrant, asymptomatic intradialytic BP changes induce harmful effects to HD patients. Thus, our study was designed to evaluate factors that may influence the fluid shifts in asymptomatic patents with intradialytic BP falls. But, there were no significant differences between the hydration status, presence of DM, age, sex, HT medication, HD vintage, and fluid distribution index. Only we found that ECW of patients with wide intradialytic SBP difference was significantly lower than that of patients with narrow intradialytic SBP difference. Unfortunately, it would be difficult to explain the exact meaning of our results. Nevertheless, further study should be needed to elucidate the changes of IVF and ISF absolute values and relevant factors in asymptomatic patients with wide intradialytic BP difference.

This study had some limitations. First, we used intradialytic BP difference as an index of BP fluctuation instead of other intradialytic BP variability index, such as standard deviation, absolute value of the difference between successive BP measurements and BP residual. However, this reflects more the BP drop during HD than the variability *per se* and a relation between a larger decline in ECW and BP is to be expected. If we repeated the analysis with additional BP variability metrics such as the BP residual in a random-effects model [4], we thought that our data had the increased reliability. However, practically it is extremely difficult to apply what needs to be repeated to HD patients. Second, the number of patients was relatively small, single-session measurements only and they were all from a single-center. Third, we could not show the changes of IVF and ISF absolute values during HD. Forth, bioimpedance measurements should be done after a half-hour rest and equilibration by definition; at least according to the BCM manufacturer. Notwithstanding, BCM was performed at the same time and conditions in all patients and we confirmed that patients with larger UF volumes had larger ECW fluid removed. In addition, our results may be the differences in the type of bioimpedance instrument used, how the electrodes are applied and what output information is available. However, strengths of our study include the use of absolute values of fluid distribution and demonstrating fluid shifts serially from start to 30 min after HD and comparing all data through appropriate grouping, which can enable reproducibility of our data.

## Conclusions

Patients with wide intradialytic SBP difference have the characteristics of fluid shifts in which reduction of ECW was steeper than patients with narrow intradialytic SBP difference whereas fluid shifts of ICW were lower in patients with wide intradialytic DBP difference than patients with narrow intradialytic DBP difference. This phenomenon has been confirmed from patients with narrow intradialytic SBP/DBP difference.
